# The influence of malocclusion on social aspects in adults: study via eye tracking technology and questionnaire

**DOI:** 10.1186/s40510-022-00399-3

**Published:** 2022-01-24

**Authors:** Gil Guilherme Gasparello, Sergio Luiz Mota Júnior, Giovani Ceron Hartmann, Thiago Martins Meira, Elisa Souza Camargo, Matheus Melo Pithon, Orlando Tanaka

**Affiliations:** 1 School of Life Sciences, Pontifícia Universidade Católica Do Paraná, Curitiba, Brazil; 2 Juiz de Fora Federal University, Juiz de Fora, Minas Gerais Brazil; 3 Graduate Dentistry Program in Orthodontics, School of Life Sciences, Pontifícia Universidade Católica Do Paraná, R. Imaculada Conceição, 115, Curitiba, PR 80215-901 Brazil; 4 Diplomate of the Brazilian Board of Orthodontics – BBO, Southwest Bahia State University – UESB, Jequié, Bahia Brazil

**Keywords:** Visual perception, IOTN, Age perception, Job, Eye-tracking

## Abstract

**Background:**

Smile esthetics has a strong influence on perception, attractiveness, and personal characteristics. It is unknown how malocclusions may influence the appearance of the individual's smile. This study aimed to investigate whether malocclusion affects the visual perception of esthetics, age, employability, honesty, intelligence and to meet its obligation in time in middle-aged adults. Facial frontal smiling photographs of a male and a female middle-aged adult with 3 different malocclusions were shown to and evaluated by 90 laypeople (non-dentists), divided into groups: young adults (14–44 years), middle-aged adults (45–59 years), and elders (over 60 years). The index of treatment need (IOTN) was used, and IOTN 1, 5, and 8 images were created in Photoshop using the male and female photographs. In total, 6 photographs were evaluated, 3 areas of interest (AOI)—eyes, nose, and mouth—were created for statistical comparison. The Ogama and Eye Tribe tracker were used in conjunction to measure eye tracking. A visual analog scale (VAS) was employed with a questionnaire surveying individuals’ perception of age, employability, honesty, intelligence, and ability to meet obligations. Kruskal–Wallis, one-way analysis of variance, Pearson’s—chi-squared, and Pearson correlation test were applied.

**Results:**

No statistical difference was found in complete fixation time and time until the first fixation for each AOI for eye-tracking. VAS showed statistical differences in the male and female IOTN 1 images when compared with the IOTN 5 and 8 for both models, and there was no difference between genders in the IOTN 1. As the perceived age of the model increased, the chances of getting hired decreased. Employability, honesty, intelligence, and ability to meet obligations showed higher values for IOTN 1. There were significant differences between age ranges; perception of intelligence in the female IOTN 1 model (*p* = 0.002) and IOTN 8 model (*p* < 0.001) and honesty between young adults and middle-aged adults in the male IOTN 1 and 8 images (*p* < 0.001).

**Conclusions:**

There was a difference between age groups in the perception of honesty and intelligence. A well-balanced and attractive was perceived as more youthful and attractive in both genders and may increase the chance of being selected for a job interview or being hired.

## Background

Dental appearance has been shown to influence the judgment of a person's facial attractiveness, as well as personal, and malocclusions may affect an individual's physical, social, and psychological condition and quality of life [1].

People commonly desire a youthful appearance, which stems from the social penalty that accompanies aging, such as being discriminated against, having difficulties attaining employment, and tolerating lower pay rates, as older adults are often judged as weak, dependent, and less attractive [2–4], while young and attractive persons are considered more capable, intelligent, responsible, and happy [1]. In general, those who look younger tend to benefit from stereotypes that favor the young [5].

Many patients who seek orthodontic treatment do not wish to improve masticatory function; they are instead motivated by dental esthetics [1]. The psychological and social benefits of orthodontic treatment have emerged as more significant than the benefits to oral health [6]. As the world population is in the process of aging, there is an increase in the demand for orthodontic treatment in the adult population [7], and as natural teeth remain in the oral cavity for a longer time, dental professionals should wait and observe more frequently adults and geriatric patients in the dental office [8].

Age-related workplace discrimination and its negative impact on mental and overall health are well documented in the literature [3, 5, 9], though is unclear how factors such as malocclusion affect laypeople’s perceptions of aging and esthetics and if these factors influence job or service finding. Studies assessing photographs and eye tracking are very important, particularly as many organizational representatives now review social media information (e.g., Facebook, Twitter) when recruiting and assessing job applicants [10]. LinkedIn is an additional popular resource for employers, which focuses on business connections and industry contacts for employers and working professionals and allows users to enhance their connectedness in their areas of expertise [11]; in this platform, the first contact and impression occurs through images.

Some studies have assessed different malocclusion or diastemas [12, 13], but there is a lack of knowledge regarding perceptions of middle-aged people with different Index of Orthodontic Treatment Need (IOTN) scores assessed using eye-tracking technology.

Therefore, the aim of this study was to investigate whether malocclusion affects the visual perception of esthetics and age and evaluate its influence on job finding in middle-aged adults, with the null hypothesis that malocclusion causes no difference in the perception of attractiveness, questionnaire scores, and eye-tracking among different malocclusion and subjects of different age groups.

## Methods

### Ethical approval

The study protocol was approved by the Ethics Committee of the university (registration number: 2,235,302). All participants gave written informed consent prior to the use of their data.

### Photograph preparation

**Fig. 1:**
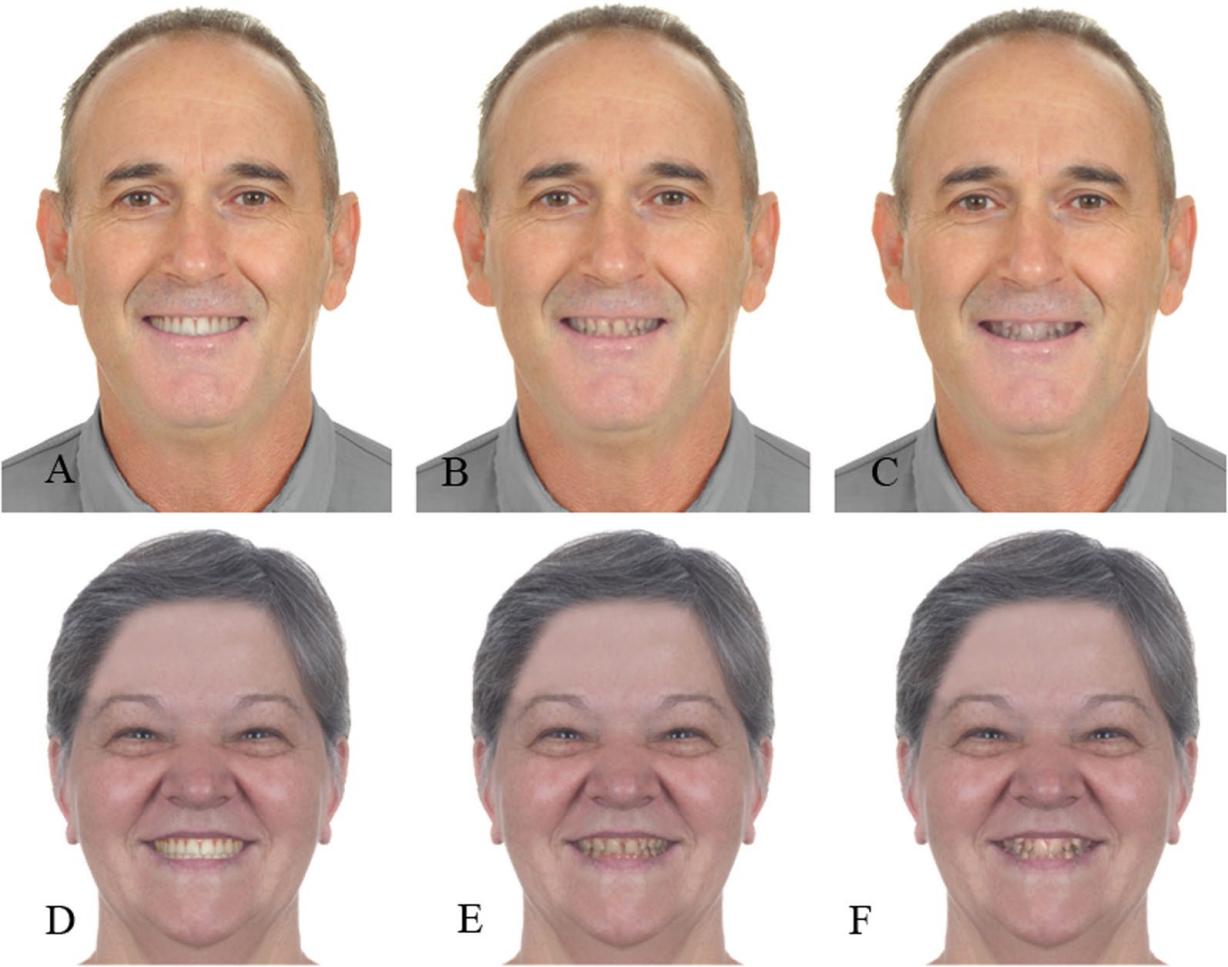
IOTN’s male and female (**A**, **D**). IOTN grades 1 (attractive without treatment), (**B**, **E**). 5 (reasonable, with diastemas and teeth not aligned and leveled); (**C**, **F**). 8 (non-attractive, with severe crowding)

Facial frontal smiling photographs of one male (56 years old) and one female (57 years old) middle-aged model without any great facial discrepancy were obtained with a digital camera (Rebel XTI; Canon, Tokyo, Japan) in an illuminated studio environment against a white background. The photographs obtained were analyzed, selected, and standardized using the Photoshop program (Adobe Systems Inc, San Jose, California). The removal of imperfections that could divert the attention of the observer, such as spots and scars, was performed, and the image was mirrored to avoid asymmetries. To create different malocclusions in the models, malocclusions corresponding to IOTN grades 1 (attractive without treatment), 5 (reasonable, with diastemas and teeth not aligned and leveled), and 8 (non-attractive, with severe crowding) were added [14] to the facial photographs using Photoshop (Fig. 1A–F). Areas of interest (AOI) comprising the eyes, nose, and mouth were established on the models’ photographs to aid statistical comparison; the AOIs were not visible in the photographs seen by the participants.

### Sample calculation

Sample calculation was performed based on the heterogeneous population of Paraná, the age group specified, an infinite population, 95% confidence level, and 10% margin of error; it was concluded that 96 subjects would be necessary for the study.

Data were collected from 109 laypeople (non-dentists), divided into 44 young adults (14–44 years), 33 middle-aged adults (45–59 years), and 32 elders (over 60 years). A total of 90 subjects had their data validated for this research, with 44 males (48%), 46 females, 45 (50%) college graduates, and 45 (50%) non-college graduates.

### Data collection

Participants were enrolled in the study based on the following inclusion criteria: absence of previous neurological and/or visual conditions; no use of alcohol, medications, or drugs that could interfere with cognitive abilities; the age of over 14 years; software calibration considered “perfect”; and all questions in the questionnaire answered. Participants who did not meet all criteria were excluded. After the application of inclusion criteria, 19 participants were excluded from the research.

Data collection took place on November 2, November 19, and December 7, 2020, in 5 cities in the state of Paraná, Brazil. The participants were randomly invited to participate, and the gender, age and educational background were asked. Eye tracking of participants was evaluated using the Eye Tribe tracker hardware (The Eye Tribe Aps, Copenhagen, Denmark) [15] in conjunction with the Ogama software (Freie Universität, Berlin) [16].

Participants were shown 21 photographs, all images presented malocclusions, although the other 15 images were from young models, in which 7 were male and 7 female, those images were discarded after the data collection. All the images were showed in a random sequence performed by the website < randomizer.org > . Among the photographs were the 6 prepared images, 3 female and 3 male, with IONTs 1, 5, and 8. The addition of more images prevented perception bias between images. Each image was visible for 5 s.

No details about the research were revealed. After clearing the inclusion criteria, the participant was directed to a silent room containing only one researcher. The participant was invited to sit in a chair positioned at a distance of 60 cm from a high-resolution Dell P2317 monitor (768 × 1366 pixels), oriented in an upright position to maintain the actual proportions of facial size, with the eye-tracking hardware positioned just below as recommended by the manufacturer. A 9-point calibration was then conducted by the software.

### Questionnaire

After the eye-tracking session, the participants were asked to answer a questionnaire. For this purpose, a Dell Inspiron 7375 touch 2-in-1 computer with the Qualtrics application was made available for use. Participants could choose to respond on their mobile devices, with the requirement that the answers would be sent while in the presence of the researcher.

The questionnaire comprised 6 questions, 4 of which were based on the studies by Henson et al. and Pithon et al. [2, 6]: “If you had to search for someone to work with you, would you consider hiring this person?”, “In your opinion, would you judge this person intelligent?”, “In your opinion, would you judge this person honestly?”, and “In your opinion, does this person seem to meet their obligations on time?”. the participants could choose to answer “yes,” or “no.”

In addition, a visual analog scale (VAS) was employed, with a score established between 0 and 100 through a digital slide bar. Scores closer to 0 indicated the image was less attractive, and scores closer to 100 indicated that the image was more attractive [17]. The participants provided their age perceptions of the analyzed image as well, with age intervals of less than 45, 45–49, 50–54, 54–59, 60–64, 65–70, and more than 70 [18]. In answering the 6 questions, the participants could work freely and answer in the order of their choice, though once a response was submitted, it could not be changed. Additional images were also scored and discarded after the completion of the study in order to avoid comparisons between consecutively arranged images. If there was any voided question, the data from the participant were excluded.

### Statistical analysis

The results obtained from the eye-tracking software and questionnaires were tabulated in Microsoft Excel and analyzed in the Statistical Package for Social Sciences version 25 (SPSS; SPSS Inc., Chicago, IL) program. One-way analysis of variance (ANOVA) was applied to analyze significant differences between images when data were normally distributed and the Kruskal–Wallis test was performed for data with non-normal distribution, including the time until first fixation, complete fixation time. ANOVA test was applied for the VAS score. Levene’s homogeneity test was applied to identify homogeneous or heterogeneous distribution. Post-hoc testing was conducted to identify statistical differences; in the event of a homogeneous population, Tukey’s honestly significant difference was used, and in the event of a heterogeneous population, Games–Howell test was applied. For the variables complete fixation on the eye, nose, and mouth and time until the first fixation on the eye, nose, and mouth, Kruskal–Wallis test was carried out to identify statistical differences. Pearson’s chi-squared test was employed to compare the different IOTNs with the variables of perception of employability, intelligence, honesty, and ability to meet obligations on time, as well as age and education. Test–test was applied to identify differences between gender. For the comparison of age ranges, all comparisons were made within the same IOTN and model to identify if the age range affected the perception of the same image.

## Results

**Fig. 2:**
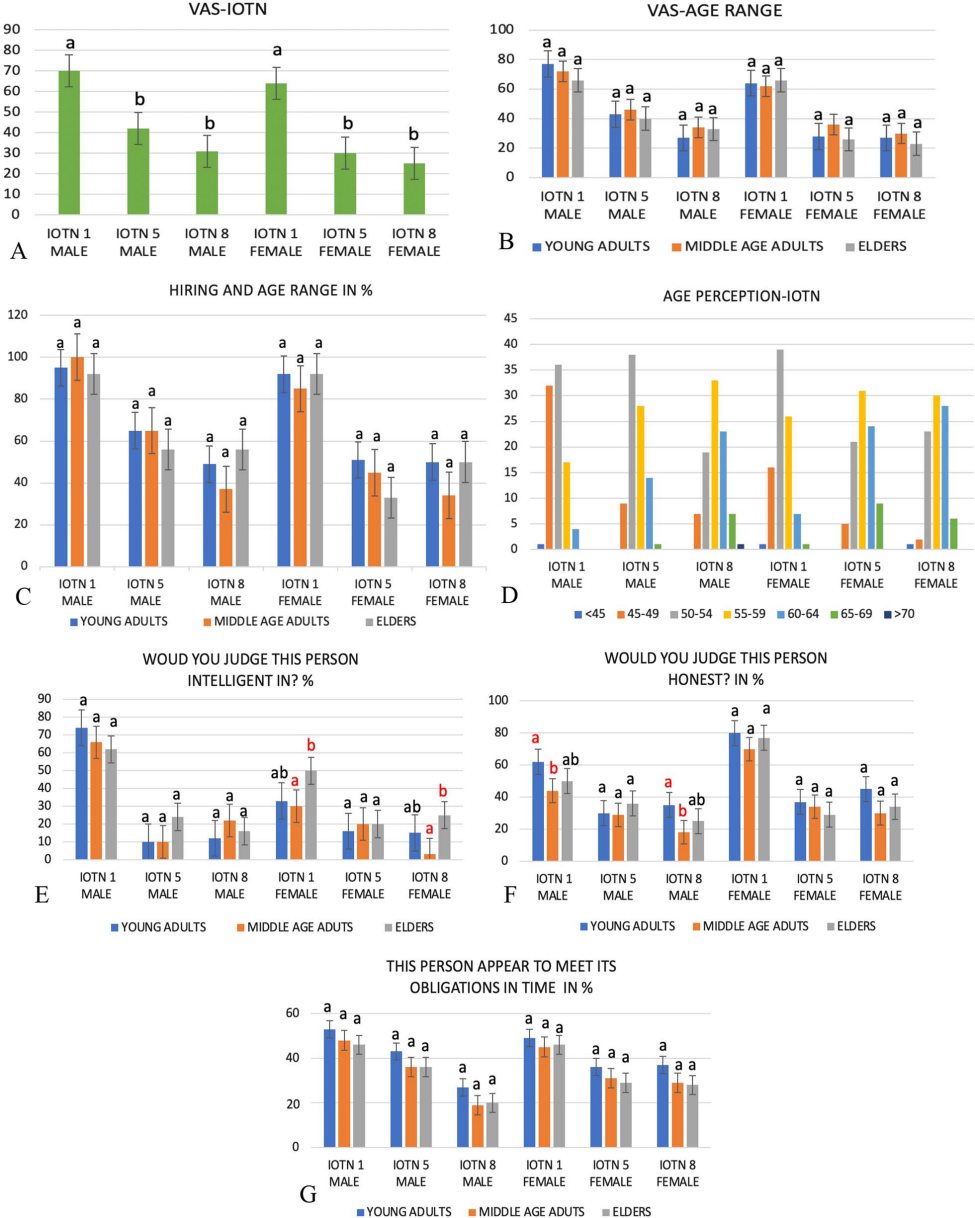
Cross-tabulation comparing **A** mean VAS and IOTN; **B** mean VAS and age range; **C** Hiring and age range; **D** Age perception and IOTN; **E** Intelligence and age range; **F** Honesty and age range; **G** Obligations in time and age range. Statistical difference p < 0.005. Different letters = Statistical difference

No statistical difference was reported in eye tracking incomplete fixation time or time until the first fixation between the AOIs of different IOTNs or between different age groups (Table 1). Images with IOTN grade 1 had higher VAS scores. Statistically significant differences were found between the male images with IOTN 1 and 5 as well as both the male and female images with IOTN 1 and 8 (*p* < 0.001) (Fig. 2A). With respect to age range, no significant difference was found between age groups when comparing the same image (Fig. 2B).

**Table 1:** Comparison using A. Kruskal–Wallis B. ANOVA

	Mean male (SD)	Mean female (SD)	Different IOTN	Age range
			Sig	Sig
A. Complete fixation time on eye (ms)	611.08 (472.73)	990.03 (1004.31)	0.945	0.313
A. Complete fixation time on nose (ms)	840.32 (844.73)	972.94 (984.53)	0.701	0.670
A. Complete fixation time on mouth (ms)	1267.95 (1009.02)	1372.00 (928.11)	0.058	0.373
A. Time until first fixation on eye (ms)	2633.86 (1205.20)	1460.43 (1245.45	0.861	0.905
A. Time until first fixation on nose (ms)	1909.35 (1574.39)	1332.84 (1455.16)	0.192	0.588
A. Time until first fixation on mouth (ms)	999.48 (1252.68)	956.32 (1227.89)	0.159	0.185
B. VAS	46.32 (28.56)	41.99 (30.38)	0.000	0.524

Statistical difference *p* < 0.05

Participants: *n* = 90

*ms* miliseconds, *SD* standard deviation

Regarding the question “Would you consider hiring this person?” a statistically significant difference was observed for both male and female images between IOTN 1 and the other IOTNs (*p* < 0.001) (Table 2). The number of participants who did not consider the models hirable increased significantly (*p* < 0.001) as their perception of the models’ age increased (Fig. 2A, Table 3). No statistical difference was found in the Pearson correlation test between age perception and participant age range, meaning that age range did not affect how the participants perceived the models’ age (Table 3). Participants who were college graduates tended to be more discerning when hiring for all the images (Table 4).

**Table 2:** Cross-tabulation and Pearson’s chi-square value

	IOTN 1 male	IOTN 5 male	IOTN 8 male	IOTN 1 female	IOTN 5 female	IOTN 8 female	*p* value
*Would you consider hiring this person?*							
Yes	86_a_	56_b_	43_b_	81_a_	40_b_	39_b_	0.000
No	4_a_	34_b_	47_b_	9_a_	50_b_	51_b_	
*Would you judge this person inteligent?*							
Yes	33_a_	17_a,b_	13_b_	62_c_	13_b_	15_b_	0.000
No	57_a_	73_a,b_	77_b_	28_c_	77_b_	75_b_	
*Would you judge this person honest?*							
Yes	67_a_	31_b,c,d_	28_d_	48_c_	28_b,d_	25_b,d_	0.000
No	23_a_	59_b,c,d_	62_d_	42_c_	62_b,d_	65_b,d_	
*Does this person appear to meet its obligations in time?*							
Yes	42_a,b_	30_a,b_	28_a,b_	47_b_	40_a,b_	25_a_	0.003
No	48_a,b_	60_a,b_	62_a,b_	43_b_	50_a,b_	65_a_	

Statistical difference *p* < 0.05

Participants 90

**Table 3:** Pearson correlation

	Pearson correlation	*p* value
Age perception and VAS	− 0.254	0.0001
Age perception and age range	0.041	0.341

Statistical difference *p* < 0.05

Participants: *n* = 90

**Table 4:** Cross-tabulation regarding education background and hiring %

			Yes	No	Pearson Chi-squared
Education	College graduate	% Hiring	45.5	57.9	0.027
	Not graduate	% Hiring	54.5	42.1	

A negative correlation was found by Pearson’s correlation in VAS score, meaning that as VAS scores increased, the perceived age decreased (*p* = 0.0001, *r* =  − 0.249). Images of IOTN 5 and IOTN 8 were perceived as significantly older by the participants compared to the images of IOTN 1, though were not significantly different from each other (Table 2). No statistical difference was found between participant age and IOTN image (Fig. 2C). The male and female images with IOTN 5 and 8 were perceived as older, although it is not possible to state a statistical difference as it was descriptive data (Fig. 2D).

In reference to the question “Would you judge this person intelligent?” a statistical difference was noted for male and female IOTN 1 images (*p* < 0.001), which were considered more intelligent at first glance when compared with the other IOTN images (Table 2). There was a difference between male and female IOTN 1 models, with the female IOTN 1 model considered more intelligent.

When the question “Would you judge this person honest?” was considered, a statistical difference was found between the male model with IOTN 1 when compared with the other IOTNs (*p* < 0.001), with the IOTN 1 male considered more honest (Table 2). Regarding the variable of meeting obligations on time, a statistical difference was found in IOTN 5 and 8 images for both genders, with the participants not considering these models as able to meet obligations on time (*p* = 0.03) (Table 2).

Middle-aged adults and elders showed significant differences for the variable of intelligence in the female IOTN 1 model (*p* = 0.002) and IOTN 8 model (*p* < 0.001), with older adults judging these models as more intelligent and honest; there was a significant difference between young adults and middle-aged adults in the male IOTN 1 and 8 images (*p* < 0.001), with middle-aged adults considering these models less honest (Fig. 2E, G).

When comparing between genders, there were no differences between the male and female models for all variables evaluated (Table 5).

**Table 5:** Comparing sex with t-test

	*p* Value
Complete fixation time on eye	0.060
Complete fixation time on nose	0.534
Complete fixation time on mouth	0.436
Time until first fixation on eye	0.434
Time until first fixation on nose	0.098
Time until first fixation on mouth	0.800
VAS	0.088
Age range	0.713
Would you consider hiring this person?	0.135
Would you judge this person intelligent?	0.321
Would you judge this person honest?	0.604
Does this person appear to meet obligations on time?	0.413
Age perception	0.203

Statistical difference *p* < 0.05

Participants: *n* = 90

## Discussion

This study explored malocclusion and how it affects the perception of age in Caucasian male and female middle-aged models and as well as social aspects such as employability, intelligence, honesty, and ability to meet obligations on time. For this study, no reliability test was performed because the aim of the study was to investigate the impact of malocclusion at first glance; if reliability testing was applied, the participant would be required to observe the same images twice.

Apparent age can determine how a person is treated by society. Many models of age-related facial changes have been proposed, most of them involving the morphological evolution of the face over time [19–21]. Once was thought the gingival biotype could be correlated with the different types of Angle’s classification of malocclusion and could interfere in esthetics, dimension, and malocclusion, it was dismissed in a study. [22]

Malocclusion and dental esthetics affect personal well-being, social interactions, and physical, social, and psychologic functioning, with a resulting effect on a person's quality of life [1, 23]. The IOTN rating system is widely used to determine malocclusion treatment needs owing to its efficiency and practicality [14]. We decided to use IOTN grades 1 (close to ideal), 5 (borderline need for treatment), and 8 (definite need for treatment) based on De Oliveira et al., who compared these 3 IOTNs between laypeople, orthodontists, and general dentists [13], and the reliability of the IOTN for studies using photographs was confirmed by Malik et al. [24].

The study’s methodology was based on the studies by Pithon et al. and Henson et al. [2, 6]. Studied athletic performance, popularity, leadership capability, and academic performance, and their results showed that in addition to the esthetic benefit of orthodontics, there are also social benefits, which confirms the findings of this study, in which the models with well-aligned teeth had better responses regarding employability, honesty, intelligence, and ability to complete obligations on time. The question “Would you consider hiring this person?” had an observed discrepancy, with the IOTN 1 male and female receiving more favorable ratings, increasing their chance of attaining a desired job or service, (*p* < 0.001) than IOTN 5 and 8 models. Age perception may affect hiring decisions; the number of participants who negatively viewed employability increased significantly as their perception of the models’ age increased.

Similar results regarding job seeking, honesty, intelligence, and ability to meet obligations on time were found [2, 25], and both studies showed statistical differences in hiring and intelligence in favor of well-balanced and aligned teeth. These findings disagree with our study, in which a statistical difference was recorded for all variables in favor of IOTN 1. This discrepancy may be due to differences in study designs, as in the studies by Almedlej et al. and Piton et al., the participants completed the study in pairs; in our study, the methods were individualized, and additional images not used for the research were shown to prevent bias. After data collection, images that were not used were discarded.

The results of the present study showed that the male with IOTN 1 appeared to be more intelligent, and both the male and female with IOTN 1 were more attractive. These results are consistent with Eli et al. [1], whose study asked participants to evaluate photographs and report their first impression of the esthetic, social, and professional aspects of subjects with intact and nonintact dentitions and found that those with intact dentition achieved the highest scores.

Our results showed that the higher the VAS scores, the lower the perception of aging, indicating that youthful images received higher scores for attractiveness. In addition, our results showed that malocclusion may interfere with the perception of age. IOTN 5 and 8 images were judged as older. During a job interview, this may have an impact on employers’ perception of employability [9].

A previous study revealed that some people may alter their physical appearance to portray a more youthful image in a job interview [9]. As photographs are required for the social media and online business connections of working professionals, this explains why we chose to conduct a photographic survey using eye-tracking and a questionnaire to evaluate what causes the perception of a youthful model.

Eye-tracking studies had gained attention in the investigation of several themes in dentistry. Tanaka et al. compared the perceptions of laypeople with dentistry students regarding diastemas [12]. De Oliveira et al. revealed differences in the perception of IOTNs between dentists, orthodontists, and laypeople [13]. Liao et al. used eye-tracking and tasks to identify age-related facial cues [26]. In our study, we explored different IOTNs to investigate how malocclusion may affect social perceptions and interfere with getting a job. Even with no differences recorded in eye tracking, the investigated social aspects showed differences in favor of IOTN 1 for both male and female models.

Many studies have compared the opinions of different groups, such as laypeople and dentists [1, 13, 26–29], while others have only assessed one group [3, 30, 31]. In the present study, we chose to include only laypeople, though divided the sample into different age ranges, including young adults (15–44 years), middle-aged adults (45–59 years), and elders (> 60 years) based on information from the World Health Organization that divides the population into these age ranges [32].

Several studies have compared different age groups. An et al. [33] reported differences in perception, satisfaction, and happiness between these age ranges. Lacerda-Santos et al. described significant differences in smile attractiveness based on different buccal corridor sizes between young laypersons between 15 and 19 years of age and laypersons over the age of 65 years; laypersons over the age of 65 years made a less critical judgment of smile attractiveness [34]. These results do not align with the results of the present study, in which only small differences were noted between middle-aged adults and elders. Sriphadungporn and Chamnannidiadha reported that age had an impact on the perception of smile esthetics [35], which also disagrees with our findings, as no significant differences were found between different age ranges. Differences were only found between different levels of malocclusion.

Chen et al. [33] and Pithon et al. [36] found that young people were critical when analyzing esthetics, which was different than the results of our study. The result that highlighted this difference occurred in the group of participants who had graduated college, to whom employability changed based on age, though not based on IOTN.

One concern of this study was about the sample, once it was divided according to age range, the power of the test could decrease. The sample calculation was held with 90 people, then they were divided into a group of 30. The sample size of cases is 540, as the 90 people evaluated different photographs (which generated the dependent variables for both eye-tracking and questionnaires). The calculation of sampling error is 540, there were 180 cases for each group, which compound a sample error of 5% with a coefficient level of 90%, decreasing the error rate of the study.

Some limitations can be observed in this study, as some participants were excluded from the study due to not fulfilling all the inclusion criteria, such as not answering all the questions or not completing the software calibration. The considerations on which hiring decisions, honesty, and intelligence are based are not limited to photographs. Moreover, the IOTN’s images were added in the models based on a previous study, some discrepancy regarding the shade of colors may be found. In addition, analyzing only male and female faces may influence the full face, not only the malocclusion of the models. However, the statistical difference was found, and we can conclude that the appearance of teeth can play an important role, influencing future employers during the screening of candidates and laypeople when seeking a professional.

## Conclusions

The null hypothesis cannot be accepted, as age-related differences were seen in the perception of intelligence between middle-aged adults and elders and in honesty between young adults and middle-aged adults. In addition, the perception of age influenced the perception of esthetics and employability. A well-balanced and attractive smile was perceived as more youthful and attractive in both genders and may increase the chance of being selected for a job interview or being hired.

## Data Availability

The datasets used and/or analyzed during the current study are availablefrom the corresponding author on reasonable request.
